# 3C and 3C-based techniques: the powerful tools for spatial genome organization deciphering

**DOI:** 10.1186/s13039-018-0368-2

**Published:** 2018-03-09

**Authors:** Jinlei Han, Zhiliang Zhang, Kai Wang

**Affiliations:** 1 Key Laboratory of Genetics, Breeding and Multiple Utilization of Corps, Ministry of Education, Fujian Provincial Key Laboratory of Haixia Applied Plant Systems Biology, Center for Genomics and Biotechnology, Fujian Agriculture and Forestry University, Fuzhou, Fujian China; 2 National Engineering Research Center of Sugarcane, Fujian Agriculture and Forestry University, Fuzhou, China

**Keywords:** Chromosome conformation capture (3C), Topologically associating domains (TADs), Hi-C, C-technologies, Chromosome territory

## Abstract

It is well known that the chromosomes are organized in the nucleus and this spatial arrangement of genome play a crucial role in gene regulation and genome stability. Different techniques have been developed and applied to uncover the intrinsic mechanism of genome architecture, especially the chromosome conformation capture (3C) and 3C-derived methods. 3C and 3C-derived techniques provide us approaches to perform high-throughput chromatin architecture assays at the genome scale. However, the advantage and disadvantage of current methodologies of C-technologies have not been discussed extensively. In this review, we described and compared the methodologies of C-technologies used in genome organization studies with an emphasis on Hi-C method. We also discussed the crucial challenges facing current genome architecture studies based on 3C and 3C-derived technologies and the direction of future technologies to address currently outstanding questions in the field. These latest news contribute to our current understanding of genome structure, and provide a comprehensive reference for researchers to choose the appropriate method in future application. We consider that these constantly improving technologies will offer a finer and more accurate contact profiles of entire genome and ultimately reveal specific molecular machines govern its shape and function.

## Background

During the higher eukaryotic cell cycle, the spatial volumes of each chromosome are not random but are organized into specific patterns, in which individual chromosomes occupy defined, mutually exclusive regions of the nuclear volume that represent a structural unit referred to as a chromosome territory (CT) [1–4]. With extensive effort, the spatial organizations of individual chromosomes and the entire genome, with resolutions down to 1kbp, have been described [5–12]. It has now been widely accepted that genome architecture is a crucial aspect of gene regulation and genome stability [1, 5, 13–20] because the highly ordered chromatin arrangement facilitates communication between genes and their regulatory elements [21–26].

Early studies of genomic conformation were largely based on cytological techniques, such as fluorescence in situ hybridization (FISH), which allows direct evaluation of the proximity between genetic loci using probes. Observations of genome architecture by FISH have revealed the existence of CTs [27–29], looping out from CTs [30–32], and the tendency for clustering of active chromatin domains [33, 34]. While this method has been a widely used tool to study topography of chromosomes or DNA fragments of interest in individual cells, and allow us to determine how the chromosomes are organized by directly viewing their position with microscopy [35, 36]. However, technical limitations such as low throughput, low resolution and probe sequence specificity make it unsuitable for elaborate genome-wide studies of chromosomal topology [37–39]. Recently, chromosome conformation capture (3C) and 3C-based techniques using high-throughput sequencing data have emerged as powerful tools to reconstruct the spatial topology at regional, whole chromosome and genome levels [40–45]. These techniques have become the most effective way to elucidate the functional impact and the potential mechanisms establishing and maintaining spatial genome organization. In this review, we describe and compare the methodologies used to study genome architecture, with an emphasis on recently developed key approaches including 3C and its derivatives. We discuss the crucial challenges facing current 3D studies based on 3C technologies and the direction of future technologies to address currently outstanding questions in the field.

## The methodologies of 3C and 3C-derived technologies

The strategy of 3C to discover genomic architecture is based on quantifying the frequencies of contacts between distal DNA segments in cell populations [46]. In contrast to cytogenetic approaches, 3C-based genomics strategies yield incomparable information-rich data describing genome topology at the genome-wide level, enabling more systematic genome topology studies at a higher resolution and throughput and providing deep insights into genome architecture and its impact on genome function. Thus, 3C technologies are revolutionizing our ability to explore genome organization from specific loci up to the whole genome.

**Fig. 1:**
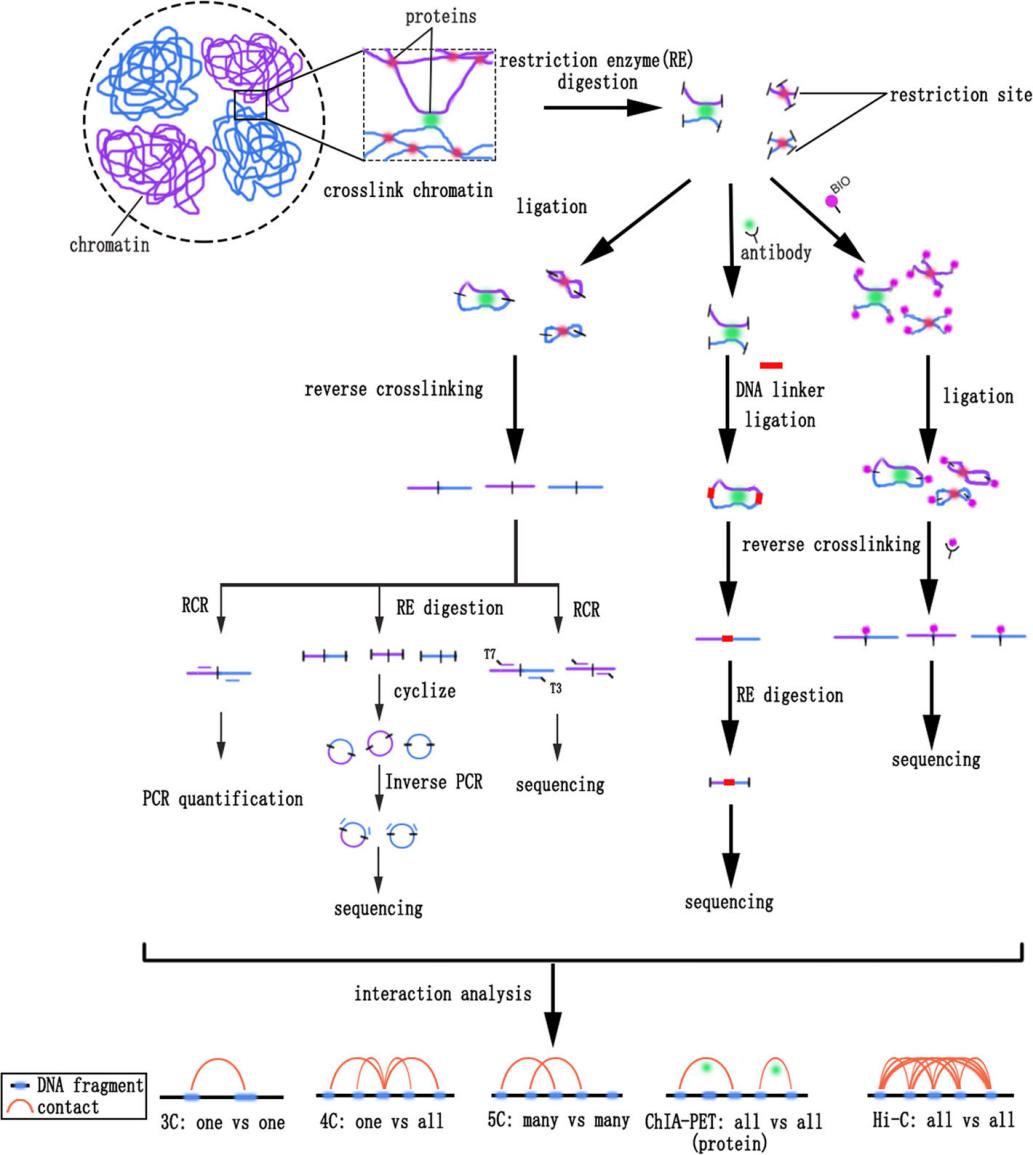
Strategy overview of 3C-based methods

The principal steps of 3C and 3C-based experiments are theoretically similar and have following principal steps: crosslink chromatin using a fixative agent in solution, most often formaldehyde, to create covalent bonds between DNA fragments bridged by proteins; isolate and digest the chromatin using a restriction enzyme such as *Hind*III [46], *Bgl*II [47], *Eco*RI [48], *Aci*I [49], or *Dpn*II [50, 51] at a low concentration to create pairs of crosslinked DNA fragments that are distant in linear distance but close in space; re-ligate the sticky ends of crosslinked DNA fragments to form chimeric molecules; reverse the crosslinks to obtain 3C templates; and finally, interrogate the rearranged DNA fragments by PCR or sequencing technologies (Fig. 1). Eventually, 3D conformations at the regional, chromosome and whole-genome levels can be inferred by calculating the number of ligation junctions between genomic loci (Fig. 1). To describe in more detail and to facilitate comparisons among different methods, we describe current 3C and 3C-based approaches below.

## Chromosome conformation capture (3C)

3C technology was developed to detect ligation junctions by PCR followed by gel electrophoresis (Fig. 1) [45]. The first 3C assay inferred the 3D conformation of yeast chromosome III and showed that it forms a contorted ring [46]. Next, this method was adapted for mammalian systems. 3C technology confirmed the existence of chromatin loops, which confer spatial contact between DNA fragments such as regulatory DNA elements and their target genes [47, 52–57] or the start and end of a gene [58]. Remarkably, this chromatin loop is dynamic based on transcriptional state changes [48, 58], implying that this structure is associated with genomic function.

**Fig. 2:**
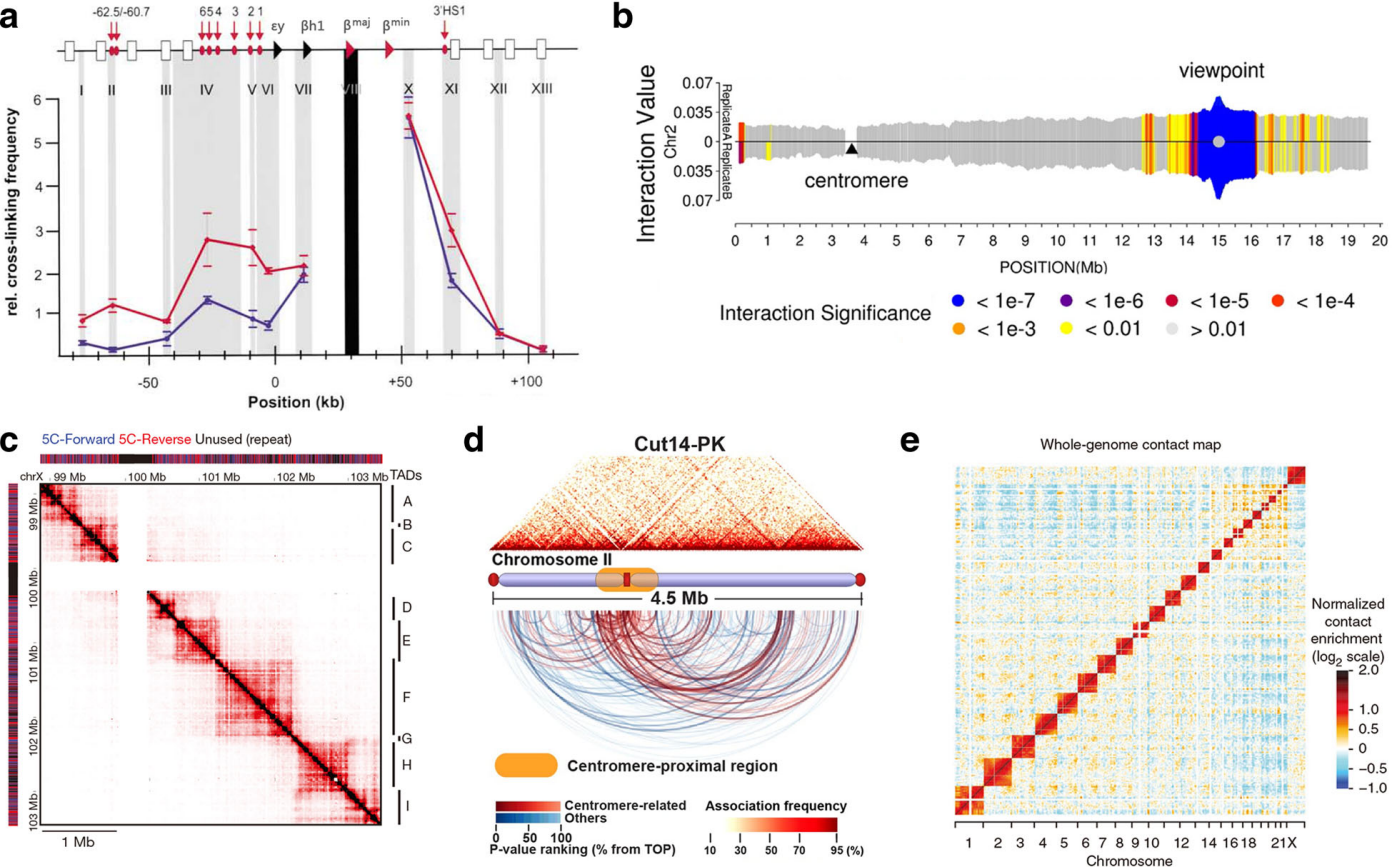
Representative output of 3C, 4C, 5C, ChIA-PET and Hi-C analysis. **a** A profile of 3C experiment for the murine β-globin locus showing looping and interaction between the Locus Control Region (LCR) and the expressing β^maj^ gene (reproduced from [47] with permission from Elsevier, ©2002). The murine β-globin locus contains hypersensitive sites (HS, red arrows and ellipses), an LCR being comprised of 5’HS1–6, globin genes including εy, βh1, β^maj^ and β^min^ (triangles), and olfactory receptor (OR) genes (white boxes). The x-axis represents position in the locus, and y-axis represents relative cross-linking frequency for the β^maj^ gene (black shading) with the rest of restriction fragments (gray shading). In erythroid cells, β^maj^ is active (red line), and the LCR come in close spatial proximity with the gene. However, the gene is silent in brain cells (blue line), and no such situation is observed. In 3C assay, primers are designed for restriction fragments of interest. Then, the spatial information between restriction fragments (one vs one) can be achieved by assessing the amplification efficiency. **b** 4C interactome of *FIS2* gene on chromosome 2 in *Arabidopsis* (reproduced from [18] with permission from CC BY 2.0 license). *FIS2* is defined as a viewpoint, and the genome is queried for positions that contact this site in space (one vs all). The results showed that chromosomal interactions have been centred around the viewpoint. **c** Interaction map of 5C assay for the 4.5-Mbp region containing *Xist* in undifferentiated mouse embryonic stem cells (reproduced from [75] with permission from Nature Publishing Group, ©2012). 5C analyses showed discrete self-associating chromosomal domains occurring at the sub-megabase scale (TADs A–I). 5C experiment requires a mix of 3C templates and thousands of primers (5C-Forward and 5C-Reverse) to allow concurrent determination of interactions between multiple fragments (many vs many). **d** Visualization of ChIA-PET associations mediated by Cut14-Pk (condensin) on chromosome II in fission yeast (reproduced from [139] with permission from Nature Publishing Group, ©2016). ChIA-PET offers the results of chromatin interactions exclusively to those fragments bound by protein of interest (all vs all mediated by specific protein). **e** Heat maps of Hi-C interactions among all chromosomes from human lymphoblast. Interaction matrix of the genome (all vs all) is built with bin size of 1Mbp (reproduced with permission from Nature Publishing Group, ©2011)

Although 3C provides a method for visualizing the genome at high resolution, some shortcomings remain, including the requirement for PCR primers designed to amplify regions of interest. For this reason, 3C can be used only to detect spatial relationships between known DNA sequences. Obviously, the “one versus one” (Fig. 2a) throughput of this method limits its application to genome-scale assays. In addition, it can detect contact only in a limited range (not exceeding a few hundred kilobases) [59]. To overcome these limitations, several 3C-derived methods have been developed to generate higher throughput chromatin interaction data.

## Chromosome conformation capture-on-Chip (4C)

4C technology was developed by combining 3C with microarray [60, 61] or, more recently, next-generation sequencing (NGS) technologies [62, 63]. This method is able to assess chromatin interactions between one genomic locus of interest (referred to as bait or viewpoint) and all other genomic loci (one versus all) (Fig. 2b). In 4C experiments, small DNA circles are created by cleaving with a second restriction enzyme and re-ligating 3C DNA templates. Then, inverse PCR using bait-specific primers is applied to amplify any interacting fragments. Finally, the interacting fragments are evaluated using microarrays or NGS (Fig. 1).

4C was originally applied to elucidate the DNA contact maps of the β-globin and *Rad23a* genes [60], which showed that the housekeeping gene *Rad23a* tends to interact with other active regions on the chromosomes and that its contact maps were conserved in various tissues [60]. By contrast, the contact maps for the erythroid-specific gene β-globin changed depending on its expression status. More specifically, β-globin contacts other active regions in erythroid cells, whereas it contacts inactive regions in the fetal brain, where this gene is silent. Subsequently, a series of 4C experiments were carried out and have shown changes in gene regulation and interaction profiles during differentiation and development [64] and that chromosome conformation is relatively stable in a given cell type [65]. Furthermore, 4C has also been used to identify chromosomal rearrangements [66] and uncover disease mechanisms [67].

4C technology is an excellent strategy to survey the DNA contact profile of specific genomic sites. However, it is worth noting that the amplification of GC-rich fragments by inverse PCR during 4C library construction is inefficient, resulting in biases in the interaction profile [68]. In addition, it is not possible to differentiate PCR duplication in 4C data.

## Chromosome conformation capture carbon copy (5C)

Another variant of 3C is 5C [69, 70]. It is analogous to 3C technology but is a “many versus many” method (Fig. 2c), allowing the simultaneous detection of millions of interactions through the use of thousands of primers in a single assay. The main difference between 3C and 5C is the strategy for primer design. 5C primers have a universal sequence (usually T7 and T3) appended to the 5′ ends. This change, combined with multiplex PCR amplification and sequencing, allows researchers to detect contact events within a particular locus (Fig. 1). Thus, in contrast to 3C, 5C has a higher throughput and a lower bias. The improvements due to this higher-throughput scale have been demonstrated by its application in the study of the human β-globin locus [69], human α-globin locus [71], and human HOXA–D gene clusters [72–74]. In addition, 5C provided the first evidence of the existence of topologically associating domains (TADs) by X chromosome analysis [75]. However, 5C is still limited in terms of the size of the genomic region that can be assayed because of the DNA sequence requirement of interested regions, as well as the quantitatively inestimable PCR duplication.

## Chromatin interaction analysis by paired-end tag sequencing (ChIA-PET)

Another 3C-based technology is ChIA-PET, which combines chromatin immunoprecipitation (ChIP) with 3C-type analysis to study genome-wide long-range chromatin interactions bound by one specific protein [76–78]. The key features of ChIA-PET technology are that the interaction sites are enriched by ChIP using a specific antibody after chromatin digestion, as in a ChIP experiment. Then, DNA sequences tethered together and to the protein of interest are connected through proximity ligation with oligonucleotide DNA linkers, the sequence of which contains restriction sites for digestion in the next step (Fig. 1). After high-throughput sequencing and bioinformatics analysis, an interactome map of the specific protein binding sites is achieved (Fig. 2d). Thus, ChIA-PET has been applied efficiently to study sites bound with specific transcription factors [76, 78, 79]. For example, some proteins, such as RAD21, SMC3, CTCF and ZNF143, that are important for the formation of a 3D chromatin structure were discovered with ChlA-PET analysis [80]. Another advantage is that ChIA-PET has relatively low levels of library complexity compared with other 3C techniques; therefore, interactions that are identified with an extremely low number of reads are usually considered significant [76, 78]. Recently, an improved method, HiChIP was developed and it can improve over 10-fiold of the yield of chromatin interacting reads but with 100-fold lower requirement than that of ChIA-PET [81]. Results generated from HiChIP of cohesin achieved multiscale genome intact map with greater signal-to-background rations than that of in situ Hi-c [81]. More important is that the sustain high-confidence results are achieved from low cell number input, which will facilitate the investigation of chromatin conformation at cell- or tissue-specific aspects.

## Hi-C

The development of high-throughput sequencing technology promoted the emergence of a series of “all versus all” methods (Fig. 2e). Of these, Hi-C was the first to be developed that does not depend on specific primers and generates genome-wide contact maps [82, 83]. In Hi-C experiments, the first step is to generate contact segments as with 3C, but the procedure is slightly different from that described above (Fig. 1). After restriction enzyme digestion, the sticky ends are filled in with biotin-labeled nucleotides followed by blunt-end ligation. The expected contacting DNAs are sheared and then purified in a biotin pull-down experiment using streptavidin beads to ensure that only biotinylated junctions are selected for further high-throughput sequencing and computational analysis (Fig. 1).

The strategy of Hi-C data analysis is thus different from above methods due to the massive parallel NGS data obtained. The basic data analysis typically involves the following aspects [84–86].

### Read mapping

Paired-end reads are independently aligned to the corresponding reference genome. Given that effective sequence fragments used for sequencing are normally chimeras, which come from two or more chromatin loci, massive reads are discontinuous in the genome [87]. Considering this situation, various methods have been proposed to improve data-use efficiency during the mapping stage, including a pre-truncation process [88], iterative mapping [89], or allowing split alignments [87].

### Read filtering

First, the mapping results are filtered at the level of reads and fragments, and only reads with information about chromatin conformation are included. Generally, valid reads are a limited distance from the nearest restriction site, and valid pairs will fall within distinct restriction fragments, which correspond to an interaction between DNA fragments. Then, the remaining read pairs are further filtered to discard PCR duplicates. Here, all uninformative reads are excluded.

### Establishment of the contact matrix

For this step, the genome is divided into non-overlapping bins, each filtered read pair is assigned to a specific bin pair, the count of read pairs in the corresponding bin pair aggregated, and eventually, a contact matrix is created. Rows and columns of the contact matrix represent bins across the genome, and each entry contains the number of read pairs that reflects bin–bin interactions.

### Normalization

Because of biases, such as GC content, the mappability, and the frequency of restriction sites, normalization (such as explicit-factor correction methods or matrix balancing-based methods) is essential to correct the raw contact matrix [89–91]. Once normalization is completed, one can generate a contact heatmap and infer genome-wide proximity information. Meanwhile, some tools have been developed to visualize Hi-C and other conformation capture data [92, 93]. These help researchers intuitively observe long-range genomic interactions.

In the original Hi-C study, two types of chromatin compartments (A and B) were identified, each of which has different functional and structural properties [82]. The former is enriched in genes and sites with active histone marks, such as H3K36me3, or DNase I hypersensitivity. By contrast, B compartments are enriched with inactive histone marks, such as H3K27me3, and contain few genes and DNase I hypersensitivity sites. Thereafter, other domain types (TAD and sub-TAD) were also identified [87, 94]. In addition, several polymer models have been showed by Hi-C data, including the fractal globule model [82], random loop model [95], dynamic loop model [96] and strings and binders switch model [97], to explain the underlying biophysical principles governing chromatin packing. For chromosome positioning, the previously observed Rabl configuration of chromosomes was confirmed once again, with the results showing clustering of the centromeres and clustering of the telomeres [98]. Due to its robust and powerful topology profiling at the genome scale, Hi-C has recently been applied extensively in genome conformation research [86, 99–102]. It is also important to mention that Hi-C can also assist in chromosomal rearrangements, genome assembly and haplotyping [16, 103–111].

Although Hi-C is a satisfactory technique for determining genome-wide chromatin interaction maps with relatively few biases compared with other existing C-methods, the acquisition of a reliable contact map with high-resolution still requires sufficient sequencing depth. Ordinarily, there is a direct relationship between mapping resolution and sequencing depth for Hi-C assay [82]. For example, generating contact profiles with resolution from 40 kbp to 1 kbp in the human genome requires hundreds of millions to multiple billion paired-end reads [112]. Thus, the cost and computational resources are certainly prohibitively expensive for most laboratories, which has seriously dampened the popularity of Hi-C.

## Other 3C-based strategies

In addition to the above techniques, other seminal adaptations of 3C protocols have been developed for assessing proximity events between distal genomic elements. For example, UMI-4C uses a unique molecular identifier (UMI) to derive quantitative and targeted chromosomal contact profiling [113]. In this approach, initial 3C ligation products are sonicated, and one end of each sonicated fragment is ligated to a sequencing adapter, generating a UMI. Using a bait-specific primer and a universal adaptor primer, physical linkage fragments can be amplified, sequenced and quantified. UMI-4C can eliminate PCR duplicates during data analysis based on the UMI and allows multiple baits (with a suggested number of 20–50 baits) to be selected [113].

Capture-C combines 3C, oligonucleotide capture technology and NGS to generate genome-wide contact profiles from hundreds of selected loci at a time [114]. In this method, 3C DNAs are sonicated, and paired-end sequencing adaptors are added. The resulting library is then enriched for junction fragments of interest by hybridization to biotinylated capture probes and streptavidin pull-down. Finally, the captured DNAs are amplified and sequenced, and the interaction maps are produced by corresponding bioinformatics methods. The Capture-C strategy can be used to enrich Hi-C libraries, and accordingly a new technique, known as Capture Hi-C (CHi-C), was developed [115]. This method enables deep sequencing of target fragments, excluding uninformative background [115, 116].

Subsequently, using an improved oligonucleotide capture strategy, Davies et al. re-designed the Capture-C protocol and developed another method, called next generation (NG) Capture-C [117]. NG Capture-C has higher assay sensitivity and resolution than Capture-C, and its sensitivity allows the analysis of weak long-range interactions. In addition, multiple 3C libraries from different cell types or different experimental conditions can be processed simultaneously in a single reaction by pooling differently indexed samples, which significantly increases throughput, removes experimental variation and allows the subtractive analysis of chromosome conformation from different samples.

Researchers have also developed DNase Hi-C technology [102]. Compared with Hi-C, the key difference is that DNase Hi-C uses DNase I rather than restriction enzymes for fragmenting crosslinked chromatin, leading to better genome coverage and resolution than Hi-C. Furthermore, the coupling of DNase Hi-C with DNA-capture technology, targeted DNase Hi-C has also been proposed and applied to characterize the 3D organization of large intergenic noncoding RNA promoters in different human cell lines [102]. Similarly, micro-C [118] and an updated micro-C XL version [119] uses micrococcal nuclease (MNase) to fragment chromatin, enabling the analysis of chromosome conformation at nucleosome resolution.

## Further considerations for 3C technologies

Researchers have been working to improve the resolution of 3C technologies to clarify the specific relationship between genomic conformation and function. However, some challenges have been encountered in this process, indicating that resolution can be affected by many factors [120]. The most important limiting factor is the selection of the first restriction enzyme, which determines the maximum resolution of 3C experiments because contacts between DNA fragments can be detected only at restriction enzyme cut sites. If two restriction enzymes, four-cutter and six-cutter, are compared, the former will yield a 16-fold higher resolution library (256 bp vs. 4096 bp) in humans. However, we should also take into account that the distribution of restriction sites is not uniform in the genome, resulting in different resolutions at different genomic regions. A further increase in resolution can be achieved by substituting restriction enzymes with MNase [118] or DNase I [102], which can cut at any site along the genome and can theoretically generate single base pair resolution. After the restriction enzyme to be used in the 3C experiment is chosen, the resolution of the contact maps is further affected by sequencing depth. When it is insufficient to explore contacts at the level of individual restriction fragments, the resolution will be determined by an appropriate bin size. In addition, research on the 3D conformation of repeat regions in the genome is difficult and is mainly because sequence information in this region is often incomplete, and thus sequencing-based 3C methods cannot effectively handle data mapping in this area. Another experimental factor that may impact the output of 3C study is the bias caused potentially by the crosslinking agent. Because crosslinking treatment inappropriately will crosslink the fibers, which are in close physical proximity rather than directly interacting [121]. Therefore, the consideration that to isolate native nuclei in an isotonic buffer to retain the native chromosome loops will properly present the native chromosome conformation [121, 122]. Moreover, comparative study of 3C-type experiments with FISH indicated that 3C-type experiments or FISH alone must be interpreted with caution when studying chromatin architecture [39], thus cross-validation of Hi-C with FISH [123] or visible data achieved with super-resolution microscope [10, 124] still need to be considered. Resemble the concept of native chromosome loops, that joint assays will present crucial information for fully understanding chromatin organization.

Theoretically, 3C experiments can capture all DNA fragments that make contact in space. However, studies have showed that capture probability present an exponential decrease as the linear distance between DNA fragments increases [82, 125]. Thus, the ligation junctions between sites that are far apart on the chromosome will be difficult to detect. Moreover, the capacity for 3C-based methods to efficiently detect simultaneous contacts between multiple genomic loci is still overrated [126]. Most recently, Beagrie et al. [127] developed an all vs. all protocol described as Genome Architecture Mapping (GAM) to dissect the nuclear architecture [127]. Instead of a strong reliance on digestion and ligation as above 3C technologies, this method combines ultrathin cryosectioning, laser microdissection, DNA sequencing, and statistical inference of co-segregation to detect interacting DNA fragments, which significantly expands our ability to explore chromatin spatial organization. Using this tool, they have succeeded in constructing the 3D chromatin structures of mouse embryonic stem cells, and unequivocally found some triplet contacts between super-enhancers [127].

Due to the heterogeneity between cells [128], it cannot be overlooked for the impact on data statistical processing regarding these data derived from millions of cells. Therefore, the established 3D genome modeling can represent only an average state of whole cell populations. Thus, one promising challenge is the development of strategies that analyze genomic 3D structure in single- or low-cell samples. Benefiting from the progresses of single-cell sequencing technology [129–131], Hi-C assays based on single or low-cell samples have been fulfilled and presented the chromatin structure diversities cell versus cell [132–138]. A prospective idea is that, by combining with other single-cell data of chromatin states, including transcriptome, DNA methylome, and histone modification, single-cell Hi-C assay will be possible to build a comprehensive picture for the interplay between chromatin folding and its states inside of single cell.

## Conclusions

C-technologies, especially Hi-C have heralded the advent of other methods that together offer tantalizing prospects for visualizing the abstract 3D structure of the genome. With the advent of other aspects such as high-throughput and long sequencing reads, single cell sequencing and other type of epigenomics data might provide us more insights on the 3D genome and reveal fundamental principles underlying genome structure and function.

## Abbreviations

3C: Chromosome conformation capture
4C: Chromosome Conformation Capture-on-Chip
5C: Chromosome conformation capture carbon copy
ChIA-PET: Chromatin Interaction Analysis by Paired-End Tag Sequencing
cHi-C: Capture Hi-C
ChIP: Chromatin immunoprecipitation
CT: Chromosome territory
FISH: Fluorescence in situ hybridization
GAM: Genome architecture mapping
MNase: Micrococcal nuclease
NG: Next generation
NGS: Next-generation sequencing
TADs: Topologically associating domains
UMI: Unique molecular identifier
